# Heart rate response during cardiopulmonary exercise in the denervated heart

**DOI:** 10.1002/ccr3.6851

**Published:** 2023-01-23

**Authors:** Ryohei Ono, Togo Iwahana, Hirotoshi Kato, Yoshio Kobayashi

**Affiliations:** ^1^ Department of Cardiovascular Medicine Chiba University Graduate School of Medicine Chuo‐ku Chiba Japan

**Keywords:** cardiopulmonary exercise test, denervation, heart rate, heart transplantation, tachycardia

## Abstract

The patients after heart transplantation usually present resting tachycardia, a slower increase in heart rate (HR) at the onset of exercise, a blunted chronotropic response to exercise in general, maximal HR being attained in the recovery period rather than at peak exercise, and a slower decline in HR after exercise.

## Graphical abstract

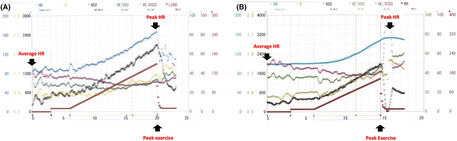

## 1 CLINICAL IMAGES

**FIGURE 1:**
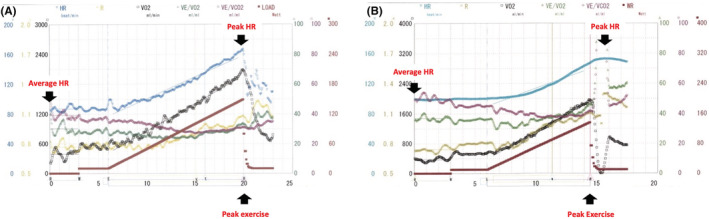
(A) The results of the cardiopulmonary exercise test (CPX) before heart transplantation, showing that the heart rate (HR) gradually increased with fluctuations during exercise, and peak HR occurred during peak exercise. The curve of HR was jagged and the average HR was normal (80 beats per min). (B) The results of CPX after heart transplantation, showing that the HR slowly increased without fluctuations during exercise, and peak HR was observed after peak exercise. The curve of HR was smooth and the average HR indicated tachycardia (100 beats per min).

A 27‐year‐old man with a history of left ventricular assist device (LVAD) implantation 8 years ago and orthotopic heart transplantation 3 years ago visited for a follow‐up cardiopulmonary exercise test (CPX). The results of CPX which was performed 1 year after the LVAD implantation showed the patient's heart rate (HR) gradually increased with fluctuations during exercise, and peak HR occurred during peak exercise (Figure 1A). In contrast, the results of CPX this time showed HR slowly increased without fluctuations during exercise, and peak HR was observed after peak exercise. The shape of the curve of HR was smooth and the average HR was 100 beats per minute (Figure 1B), indicative of tachycardia.

The heart is dually innervated by the vagal and sympathetic fibers. However, heart transplantation results in complete surgical denervation of the donor heart.^1^ Therefore, an increase in HR during exercise can be attributed to circulating catecholamines in the denervated heart.^2^ The patients after heart transplantation usually present resting tachycardia, a slower increase in HR at the onset of exercise, a blunted chronotropic response to exercise, maximal HR being attained in the recovery period rather than at peak exercise, and a slower decline in HR after exercise. HR may be influenced by the medications such as beta‐blockers, calcium channel blockers, and cilostazol. Although he was taking medications such as aspirin and immunosuppressants after the heart transplantation, these treatments would not affect this HR response. Our observation is uncommon, also in a population of heart‐transplanted patients, and it indicates particular pathophysiological conditions.

## AUTHOR CONTRIBUTIONS

**Ryohei Ono:** Conceptualization; data curation; writing – original draft. **Togo Iwahana:** Conceptualization; validation; writing – original draft. **Hirotoshi Kato:** Conceptualization; validation; writing – original draft. **Yoshio Kobayashi:** Supervision; writing – review and editing.

## FUNDING INFORMATION

The authors did not receive any funding for the present work.

## CONFLICT OF INTEREST

The authors declare no conflict of interest.

## CONSENT

Written informed consent was obtained from the patient to publish this report in accordance with the journal's patient consent policy.

## Data Availability

Not applicable.
